# Density Effect on Flame Retardancy, Thermal Degradation, and Combustibility of Rigid Polyurethane Foam Modified by Expandable Graphite or Ammonium Polyphosphate

**DOI:** 10.3390/polym11040668

**Published:** 2019-04-11

**Authors:** Hongyu Yang, Hongyin Liu, Yuping Jiang, Mingfeng Chen, Chaojun Wan

**Affiliations:** 1 College of Materials Science and Engineering, Chongqing University, Chongqing 400045, China; 20160913102@cqu.edu.cn (H.L.); 20160902037t@cqu.edu.cn (Y.J.); cjwan@cqu.edu.cn (C.W.); 2 State Key Laboratory of Coal Mine Disaster Dynamics and Control, Chongqing University, Chongqing 400044, China

**Keywords:** rigid polyurethane foam, apparent density, flame retardancy, thermal degradation, combustibility

## Abstract

The current study aims at comparatively investigating the effect of apparent density on flame retardancy, thermal degradation and combustion behaviors of rigid polyurethane foam (RPUF), RPUF/ expandable graphite (EG) and RPUF/ ammonium polyphosphate (APP). A series of RPUF, RPUF/EG and RPUF/APP samples with different apparent densities (30, 60 and 90 kg/m^3^) were prepared. The flame retardancy, thermal degradation, and combustion behaviors of each sample were investigated. Limiting oxygen index (LOI) results indicated that increasing apparent density was beneficial to the flame retardancy of all foam systems. The effect of apparent density on the enhancement of flame retardancy followed the sequence of RPUF < RPUF/APP < RPUF/EG. Thermogravimetric analysis (TGA) results showed that an increase in the apparent density can cause more weight loss in the first degradation stage and less weight loss in the second degradation stage for all foam systems. The combustion behaviors also showed significant differences. The samples with a higher apparent density showed a longer duration of heat release and higher total heat release (THR). The findings in this study demonstrated that apparent density played an important role in flame retardancy, thermal degradation, and combustion behaviors of RPUF, which must be paid more attention in the studies of flame-retardant RPUF.

## 1. Introduction

Rigid polyurethane foam (RPUF), one of the most broadly used organic thermal insulation materials, has a bright future because of its multiple merits such as low thermal conductivity, light weight, low permeability, excellent mechanical performance, and convenience construction, etc. [1,2,3,4]. However, it has significant fire hazards when used as building external wall insulation material due to its high flammability. Hence, enhancing the fire safety of RPUF is an important subject. The traditional flame retardants for RPUF were halogen-containing compounds, which can effectively increase the flame retardancy of RPUF. Nevertheless, the halogen-containing RPUF would release corrosive products and toxic gases during combustion, which are not only harmful to escape, but also poisonous to the environment. Consequently, it is imperative to research the halogen-free flame-retardant systems of RPUF [3,4,5,6,7,8]. 

A large amount of work has been done to enhance the flame-retarding efficiency of halogen-free systems for RPUF from the literature [9,10], which mainly included the introduction of flame retardants containing phosphorus [11,12,13,14], nitrogen [15,16,17], silicon [18,19,20,21], and boron [22,23,24], etc. There are two major methods for enhancing the flame retardancy of a material, which are “additive-type” and “reactive-type” methods [25,26]. In general, without purposely adjusting the dosage of foaming agent, the incorporation of many flame retardants into RPUF during the foaming process would cause changes of apparent density. Jin et al. [27] studied the mechanical and fire-proofing properties of PU/ melamine-formaldehyde (MF) foams under gradually increasing apparent density from 20.8 to 45.2 kg/cm^3^ with the increase of MF content. Yang et al. [28] studied the mechanical and fire safety of RPUF with a reactive flame retardant under varying apparent density (36.8 to 48.2 kg/m^3^). Gao et al. [29] investigated the thermal degradation and flame retardancy of RPUF modified by a novel macromolecular intumescent flame retardant (MIFR) under varying apparent density (65 to 100 kg/m^3^). Luo et al. [30] studied the morphology and properties of RPUF modified by different-sized expandable graphite (EG) under varying density from 76.42 to 116.18 kg/m^3^ with the increase of EG content. From the literature above, it can be concluded that the addition of flame retardants into RPUF would change its apparent density. Most of the reports studied the influence of apparent density (about 30–100 kg/m^3^) on mechanical properties such as compressive strength, but rarely took into account how the apparent density affects the flame retardancy, thermal degradation, and combustion behaviors of RPUF. 

Of course, in some literature, the effects of flame retardants on properties of RPUF under the same apparent density by varying the dosage of the blowing agent have also been studied. Li et al. [31] investigated the effects of EG and dimethyl methyl phosphonate (DMMP) on RPUF based on modified castor oil under the foam density at about 60 kg/m^3^. Meng et al. [32] studied the influence on APP and EG on flame retardancy and mechanical performance of RPUF at density (80kg/m^3^). Ye et al. [33] synthesized EG-poly(methyl methacrylate) composite particles and studied their influence on flame retardancy of RPUF under the same density (200 kg/m^3^). Bian et al. [15,19] studied the flame retardancy of RPUF/EG/whisker silicon oxide composites and RPUF/EG/ hollow glass microsphere composites under the same apparent density of 80 kg/m^3^. It is more persuasive for studying the effect of flame retardants on properties of RPUF in the same apparent density. Otherwise, it is difficult to differentiate the effects caused by the flame retardants and the apparent density. Therefore, understanding how the apparent density affecting the flame retardancy, thermal degradation, and combustion behaviors of RPUF is important. Bian et al. [34] reported the influence of apparent density (65 to 510 kg/m^3^) on flame retardancy of EG-filled RPUF and found that a foam with a higher apparent density had more considerable flame retardancy. Nevertheless, the studies are not comprehensive enough to understand the effect of apparent density on properties of RPUF, such as thermal degradation and combustion behaviors etc.

In this work, a series of RPUF, RPUF/EG and RPUF/APP samples with different apparent densities (30, 60 and 90 kg/m^3^) were prepared via adjusting the amount of water (chemical foaming agent). The effects of apparent density on flame retardancy, thermal degradation, and combustion behaviors of RPUF, RPUF/EG, RPUF/APP are analyzed in detail. Meanwhile, the relevant mechanisms are also discussed and speculated in this paper.

## 2. Materials and Methods

### 2.1. Materials

EG was purchased from Qingdao Tianhe Graphite Company (Qingdao, China, mesh size 80). Ammonium polyphosphate (APP) was purchased from Shifang Changfeng Chemical Company (Shifang, China, phase II, the degree of polymerization > 1000). Polyether polyol (Model: 4110), silicone surfactant (Si-Oil), 33% triethylenediamine (A33), dibutyltin dilaurate (LC) and triethanolamine (TEA) were supplied by Jiangsu Luyuan New Materials Company (Nantong, China). Polyaryl polymethylene isocyanate (PAPI) was supplied by Wanhua Chemical Group Company (Yantai, China, PM-200). Distilled water (was prepared in our laboratory). 

### 2.2. Preparation of RPUF, RPUF/EG and RPUF/APP

**Figure 1:**
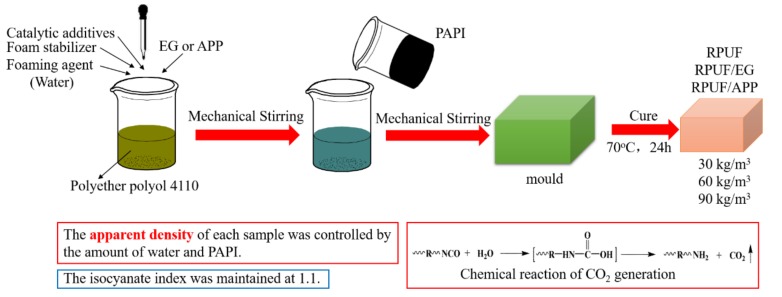
Preparation of RPUF, RPUF/EG and RPUF/APP at different apparent densities.

The RPUF samples were prepared on a laboratory scale (Figure 1) and their formulation compositions are shown in Table 1. 

The preparation steps of RPUF were as follows. Firstly, all components except PAPI were mixed in a 500 mL plastic beaker except PAPI, by a high-speed blender until obtaining a uniform mixture. Secondly, add PAPI was added into the beaker and stirred for 15 s vigorously. Thirdly, the resultant mixture was poured into a mold immediately to produce free-rise foams. In addition, then, the foams were kept into an oven at 70 °C for 24 h to obtain the polymerization reaction completely. For RPUF/EG and RPUF/APP systems, the preparation steps were similar to RPUF, except EG or APP was firstly added into PAPI before mixing with polyols and others. Cured foams were cut into specific sizes for different tests.

In this article, the contents of EG or APP in RPUF/EG or RPUF/APP systems with different apparent densities were maintained at 10 wt. % of the weight of all the raw materials. For concise description, RPUF filled with 10 wt. % of EG was shortened as RPUF/EG. RPUF filled with 10 wt. % of APP was shortened as RPUF/APP. RPUF with apparent densities of 30, 60 and 90 kg/m^3^ were shortened as RPUF30, RPUF60, and RPUF90, respectively. RPUF/EG with apparent densities of 30, 60 and 90 kg/m^3^ were shortened as RPUF30/EG, RPUF60/EG and RPUF90/EG, respectively. RPUF/APP with apparent densities of 30, 60 and 90 kg/m^3^ was shortened as RPUF30/APP, RPUF60/APP and RPUF90/APP, respectively. The different apparent densities of all samples were obtained by adjusting the amount of water in the formula. The isocyanate index was maintained at 1.1.

### 2.3. Characterization

According to ASTM D1622, the apparent densities of the foams were measured. Each sample was cut into 30 × 30 × 30 mm^3^ for the test. In addition, the final densities were obtained from the average value of three repeating tests at least.

The limiting oxygen of the foams was tested using an HC-2 oxygen index meter (Nanjing, China) and measured according to ASTM D2863. The size of each sample was 127 × 10 × 10 mm^3^. 

Thermogravimetric analysis (TGA) was performed with a TG/DSC thermal analyzer (Zurich, Switzerland). The temperature range was set from room temperature to 700 °C with a heating rate of 20 °C/min under air flow.

The combustion behavior tests were evaluated by a cone calorimeter (FTT, East Grinstead, West Sussex, UK) according to ISO 5660-1 under the heat flux of 35 kW/m^2^. The size of the specimen was 100 × 100 × 25 mm^3^.

## 3. Results

### 3.1. Flame Retardancy Analysis from LOI Test

The flame-retarding properties of RPUF, RPUF/EG and RPUF/APP at different apparent densities were characterized by determining the limiting oxygen index (LOI). 

**Figure 2:**
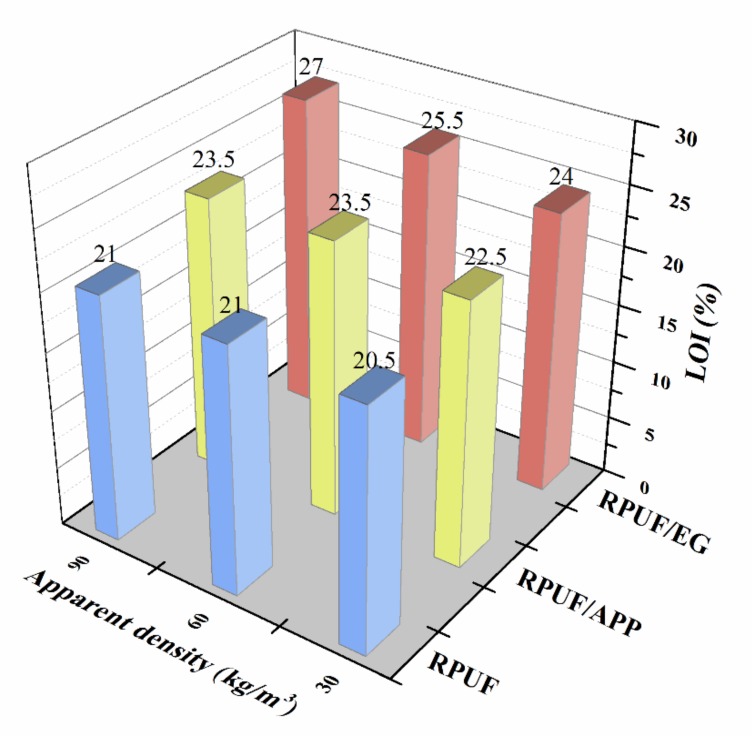
LOI values of RPUF, RPUF/EG and RPUF/APP at different apparent densities.

Figure 2 shows the effect of apparent density on the LOI behaviors of RPUF, RPUF/EG and RPUF/APP. It is conspicuous that the LOI behaviors are dissimilar from the three RPUF systems. For the pure RPUF, the LOI value of the sample at the apparent density of 30 kg/m^3^ was 20.5%, while the LOI increases slightly to 21% with the apparent density of foam increasing to 60 kg/m^3^. Nevertheless, the LOI value was not further increased with the increasing apparent density to 90 kg/m^3^. The LOI behavior of pure RPUF indicated that the increase of apparent density of foam was beneficial to the flame retardancy of RPUF, but the LOI increment is limited, which is in good agreement with the report of Bian and his coauthors [34]. For the RPUF/APP systems, the sample containing 10 wt. % of APP at 30 kg/m^3^ possessed a LOI value of 22.5%. When the apparent density of the sample increased to 60 kg/m^3^, the LOI value increased to 23.5%, while the sample with further increasing apparent density to 90 kg/m^3^ does not have a higher LOI value. The LOI behavior of RPUF/APP systems indicated that the effect of apparent density of the foam is more significant in the presence of APP compared to that of pure RPUF. For the RPUF/EG systems, the LOI value of foams increased linearly from 24% to 27% with the apparent density increased from 30 to 90 kg/m^3^, which indicated that the effect of apparent density on flame retardancy of foam in presence of EG is extremely significant. The above analysis of LOI results indicated that apparent density is an essential factor for the flame retardancy of RPUF, and the impact is more significant in the presence of APP or EG, especially EG.

### 3.2. Thermal Degradation Analysis from TG Test 

**Figure 3:**
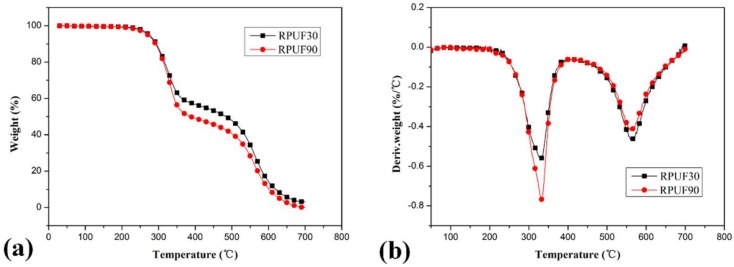
TG/DTG curves of RPUF at apparent densities of 30 and 90 kg/m^3^, (**a**). TG curves; (**b**). DTG curves.

**Figure 4:**
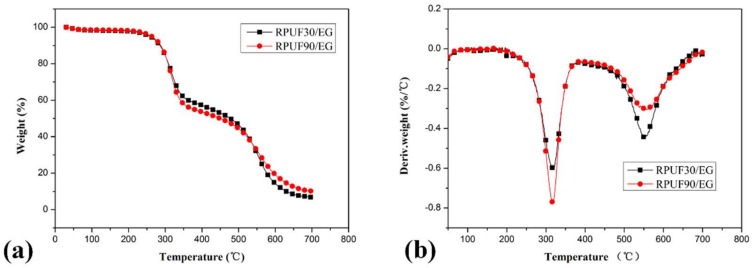
TG/DTG curves of RPUF/EG at apparent densities of 30 and 90 kg/m^3^, (**a**). TG curves; (**b**). DTG curves.

**Figure 5:**
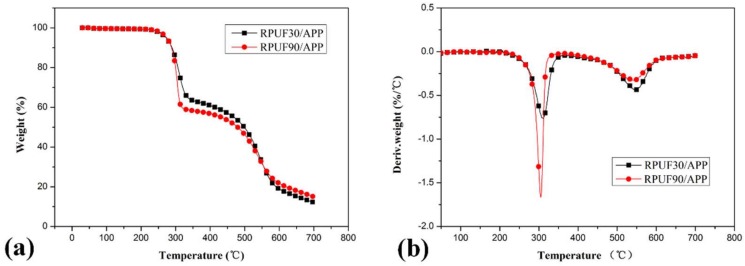
TG/DTG curves of RPUF/APP at apparent densities of 30 kg/m^3^ and 90 kg/m^3^, (**a**). TG curves; (**b**). DTG curves.

**Figure 6:**
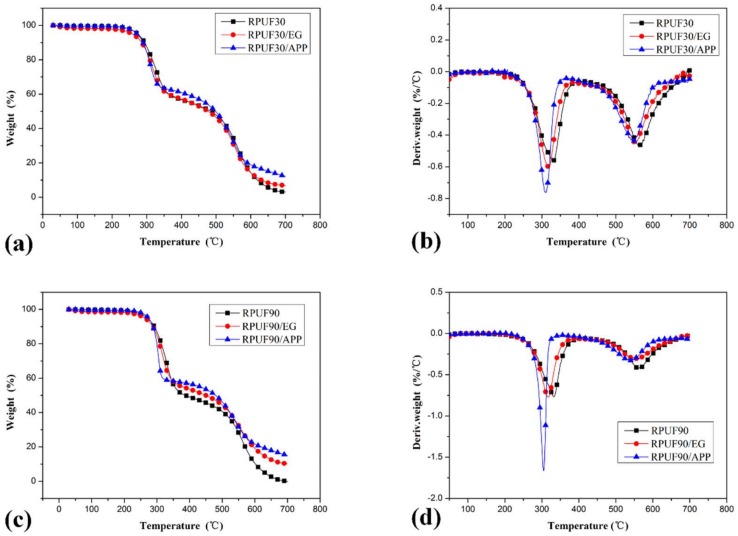
TG/DTG curves of RPUF, RPUF/EG and RPUF/APP at apparent densities of 30 and 90 kg/m^3^, (**a,b**). TG curves of RPUF, RPUF/EG and RPUF/APP at 30 and 90 kg/m^3^, respectively; (**c,d**). DTG curves of RPUF, RPUF/EG and RPUF/APP at 30 and 90 kg/m^3^, respectively.

TG and differential thermogravimetry (DTG) curves for RPUF, RPUF/EG and RPUF/APP systems at different apparent densities under air flow are shown in Figure 3 Figure 4 Figure 5 Figure 6, respectively. The corresponding data including the initial degradation temperature (*T*_initial_, the temperature at 5.0% weight loss), the maximum-rate degradation temperature (*T*_max_) of different degradation stages, weight loss at different stages and char residues at 700 °C are listed in Table 2.

From all of the Figures, it can be seen clearly that the process of thermal degradation is mainly made up of two stages, which is in good agreement at the literature [6,35]. In the first stage, polyurethane degraded into monomer precursors such as isocyanates and polyol, and then the dimerization of isocyanate formed carbodiimide at the evolution of volatiles as alcohols, CO_2_, CO, aldehydes, amines, etc. The second stage is the degradation of substituted urea (formed by the reaction of carbodiimide at water vapor or alcohol). 

From Figure 3, it can be observed obviously that there are some differences in the thermal degradation behaviors of pure RPUF samples at apparent densities of 30 and 90 kg/m^3^. Although the differences of *T*_initial_ and *T*_max_ (including *T*_max_ in stage I and stage II) between pure RPUF at 30 and 90 kg/m^3^ are relatively trivial, the weight loss of these two samples at the first and second degradation stages are quite different. The weight loss of RPUF sample at 30 kg/m^3^ in the first and second degradation stages are 41.99% and 54.83%, respectively, while that of RPUF sample at the apparent density of 90 kg/m^3^ is 50.67% and 49.49%. This phenomenon indicated that the pure RPUF at a higher apparent density (90 kg/m^3^) show more weight loss in the first degradation stage and less weight loss in the second degradation stage comparing at pure RPUF at a lower apparent density (30 kg/m^3^). In the first degradation stage, the more weight loss of RPUF at 90 kg/m^3^ may be attributed to its higher thermal conductivity, which is beneficial to the degradation of polyurethane and release of generated volatiles comparing at that of RPUF at 30 kg/m^3^. In the second degradation stage, the less weight loss of RPUF at 90 kg/m^3^ may be due to its denser structure, which is beneficial to the char formation comparing at that of RPUF at 30 kg/m^3^. From the char residue at 700 °C, both samples show limited char residues (3.18% and 0.14% for RPUF at 30 and 90 kg/m^3^, respectively). The total weight loss of RPUF at 90 kg/m^3^ is larger than that of RPUF at 30kg/m^3^, which is the combined result of weight loss in the two degradation stages. 

Figure 4 shows the thermal degradation behaviors of RPUF/EG systems. Two degradation stages of each sample also can be observed from the TG curves comparing at that of pure RPUF systems. The thermal degradation of RPUF/EG at 30 and 90 kg/m^3^ showed different behaviors. In the first degradation stage, the RPUF/EG sample at higher apparent density (90 kg/m^3^) showed more weight loss comparing at that of RPUF/EG at 30 kg/m^3^ (44.71% and 41.89% of weight loss for RPUF/EG at 90 and 30 kg/m^3^, respectively). In the second degradation stage, the weight loss of RPUF/EG at 90 kg/m^3^ (42.87%) is lower than that of RPUF/EG at 30 kg/m^3^ (50.72%). Consequently, the effects of apparent density on the two thermal degradation stages of RPUF/EG are similar to that of RPUF. However, more char residue of RPUF/EG at 90 kg/m^3^ at 700 °C (the char residue at 700 °C of RPUF/EG at 90 kg/m^3^ is 10.17%, while that of sample at 30 kg/m^3^ is 6.76%) can be observed, which caused by the combined result of different weight loss in the two degradation stages. The above analysis reveals that a higher apparent density of RPUF in the presence of EG is beneficial to the high-temperature thermal stability. 

The TG curves of RPUF/APP samples at 30 and 90 kg/m^3^ are shown in Figure 5 and the relevant data are listed in Table 2. From Table 2, it is observed that the *T*_initial_ of RPUF/APP at 30 and 90 kg/m^3^ is lower than those of pure RPUF, which may be ascribed to the influence of catalytic degradation of APP in RPUF [36]. Comparing the degradation behaviors of RPUF/APP at 30 and 90 kg/m^3^, a similar rule can be found at that of RPUF/EG systems. The sample at a higher apparent density (90 kg/m^3^) shows more weight loss (40.97%) in the first degradation stage and less weight loss (38.36%) in the second degradation stage, while RPUF/APP at lower apparent density (30 kg/m^3^) show weight loss of 36.87% and 46.11% in stage I and stage II of thermal degradation. From the char residue data, the sample at 90 kg/m^3^ show more char residue (14.94%) than that of the sample at 30 kg/m^3^ (12.14%). The TG results above indicate that the sample at a higher density in the presence of APP has higher thermal stability at high temperatures, which may be due to the catalytic char-forming effect of APP [37]. 

From the DTG curves of RPUF, RPUF/EG and RPUF/APP in Figure 3, Figure 4 and Figure 5, it can be found that the samples in all of these systems at 90 kg/m^3^ showed a higher thermal degradation rate in the first degradation stage and a lower thermal degradation rate in the second degradation stage than that of samples at 30 kg/m^3^, which indicated that the apparent density has an important impact on thermal degradation of RPUF and RPUF filled at APP or EG.

To compare the effect of EG and APP on thermal degradation behaviors of RPUF at different apparent densities, the TG and DTG curves of samples of RPUF, RPUF/EG and RPUF/APP at 30 and 90 kg/m^3^ were recombined respectively and showed in Figure 6. It can be observed clearly that the integration of EG or APP into RPUF can increase the char residue at 700 °C under the apparent density of both 30 and 60 kg/m^3^. It also can be seen from Figure 6 that the enhancement of APP or EG on thermal stability of RPUF are more significant in the systems at higher apparent density.

### 3.3. Combustion Behaviors Analysis from Cone Test

**Figure 7:**
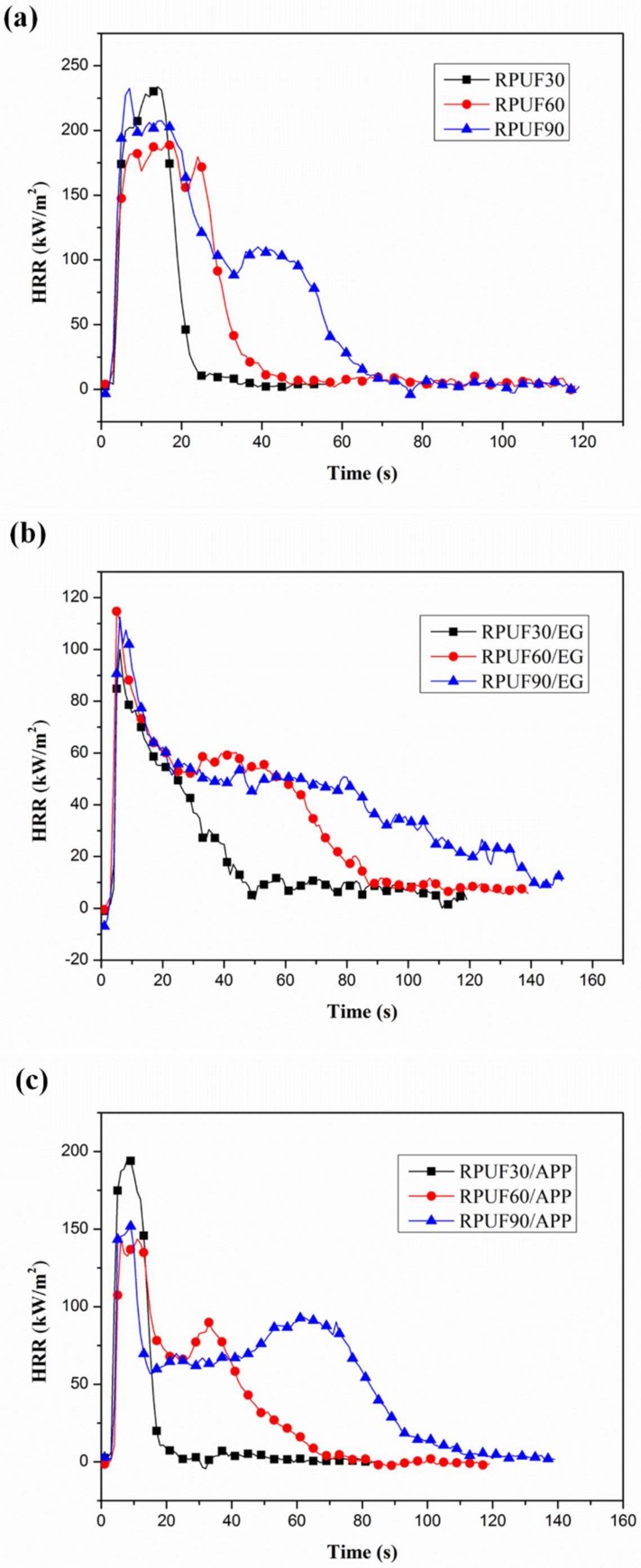
HRR curves of RPUF, RPUF/EG and RPUF/APP at different densities (30, 60 and 90 kg/m^3^): (**a**) RPUF, (**b**) RPUF/EG, (**c**) RPUF/APP.

**Figure 8:**
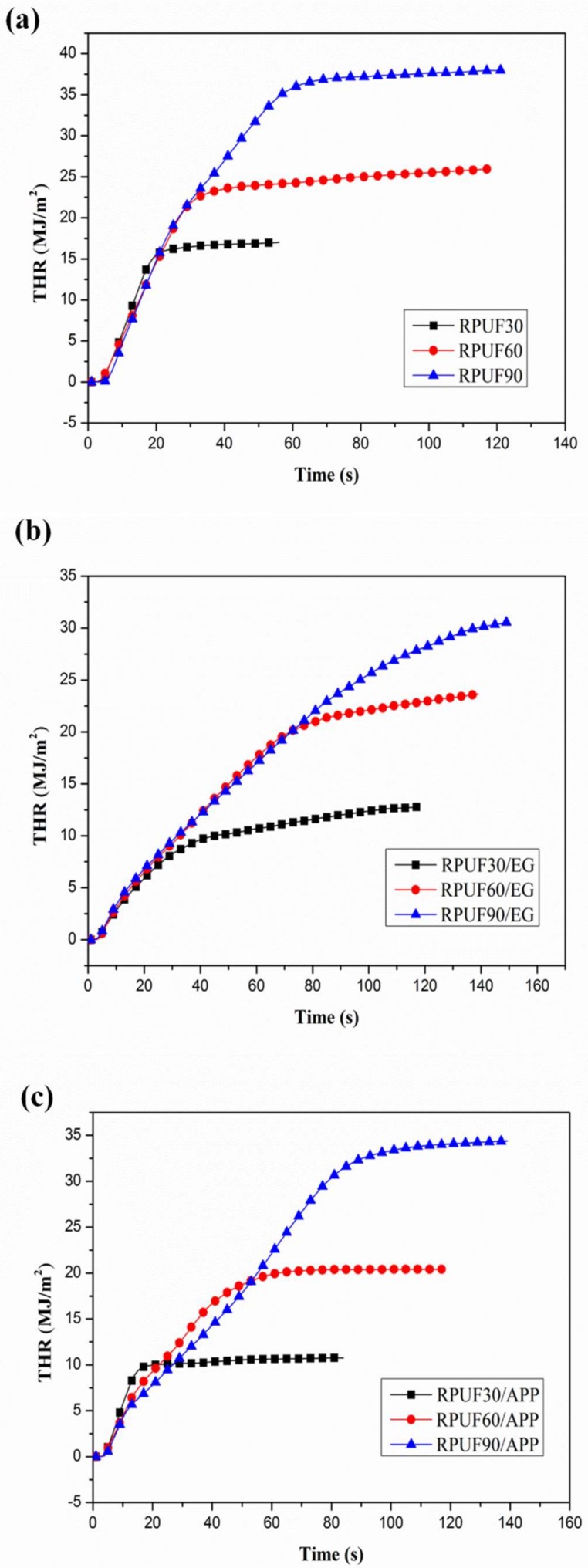
THR curves of RPUF, RPUF/EG and RPUF/APP at different densities (30, 60 and 90 kg/m^3^): (**a**) RPUF, (**b**) RPUF/EG, (**c**) RPUF/APP.

The effect of apparent density on combustion behaviors of RPUF, RPUF/EG and RPUF/APP were evaluated by cone test. Heat release rate (HRR)/THR curves for RPUF, RPUF/EG and RPUF/APP systems at different apparent densities under a heat flux of 35 kW/m^2^ are shown in Figure 7 and Figure 8, respectively. The relevant peak of heat release rate (pHRR) and THR data are listed in Table 3.

From Figure 7a, it can be seen clearly that very big differences of combustion behaviors exist between the RPUF samples at different apparent densities. The shape of HRR curves changed from a sharp peak to two peaks (a high peak appeared firstly and at a short broad peak behind) at the increase of apparent density, which is in good agreement with the report of Schartel and his coauthors [38]. The peak width of HRR curves of pure RPUF increased gradually at the increase of apparent density from 30 to 90 kg/m^3^, which can be explained as follows. The fuel of the sample at a higher apparent density is more than that of the sample at a lower apparent density under the same size (100 × 100 × 25 mm^3^). Therefore, the sample at a higher density can release more heat during the cone test and possess a longer duration of heat release so that the peak width of HRR curves increased gradually. From the pHRR values, it can be observed that the sample at 30 kg/m^3^ of density possesses a higher value (233.68 kW/m^2^). It is worth mentioning that the HRR curve of RPUF at 90 kg/m^3^ shows an obvious short and broad peak, which was caused by the charring behavior of RPUF during the burning process. Figure 7b shows the effect of apparent density on HRR curves of RPUF in the presence of EG. A sharp peak also can be observed in the HRR curve of RPUF/EG at 30 kg/m^3^, the combustion behavior characteristics also similar to thermally thin materials [38]. At the increase of apparent density of foams, the combustion behavior characteristics changed to the thermally thick charring samples. The effect of apparent density on HRR curves of RPUF/APP is shown in Figure 7c. The combustion behavior of thermally thin materials of a sharp peak also can be observed in the HRR curve of RPUF/APP at 30 kg/m^3^. The HRR curves of RPUF/APP at 60 kg/m^3^ and 90 kg/m^3^ show a higher peak and a short peak, which indicated the strong char-forming effect of APP. 

From the THR curves of RPUF, RPUF/EG and RPUF/APP at different apparent densities in Figure 8, it can be observed clearly that the THR values of all three systems increased gradually at increase of apparent densities of the foams, which also can be attributed to the increase of fuel at a higher density under the same size.

Therefore, it is necessary to compare the influence of flame retardants on combustion performance of RPUF under the same apparent density, because it is difficult to distinguish the changes occurred on the HRR curves caused by the flame retardants or the apparent density.

### 3.4. Char Residues Analysis after Cone Test

**Figure 9:**
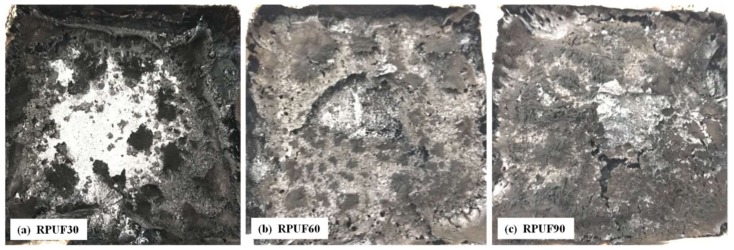
Digital images of RPUF after cone test: (**a**) 30 kg/m^3^, (**b**) 60 kg/m^3^, (**c**) 90 kg/m^3^.

**Figure 10:**
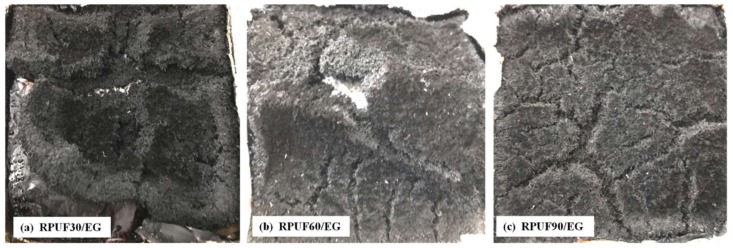
Digital images of RPUF/EG after cone test: (**a**) 30 kg/m^3^, (**b**) 60 kg/m^3^, (**c**) 90 kg/m^3^.

**Figure 11:**
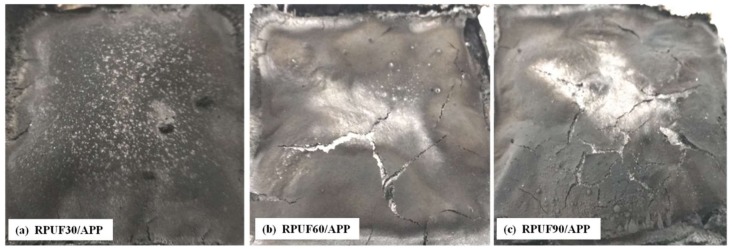
Digital images of RPUF/APP after cone test: (**a**) 30 kg/m^3^, (**b**) 60 kg/m^3^, (**c**) 90 kg/m^3^.

The digital images of char residues of RPUF, RPUF/EG and RPUF/APP systems at different apparent densities (30, 60 and 90 kg/m^3^) after cone test are showed in Figures 9, Figure 10 and Figure 11. 

From Figure 9, it can be seen that there is char residue formed after cone test for all the pure RPUF at different apparent densities and the char residue increased gradually at the increase of apparent density of foams. The char residue of RPUF at 30 kg/m^3^ was limited and not enough to cover the aluminum-foil paper, which is not difficult to understand the sharp peak occurred in the HRR curve in Figure 7a. At the apparent density of RPUF sample increasing to 90 kg/m^3^, the increasing char residue was enough to cover the surface of the sample, which can retard heat release and cause the appearance of the second short and board HRR peak in Figure 7a. 

From Figure 10, the char residues of RPUF at different apparent densities in the presence of EG can be observed clearly. All these three samples possessed more char residues comparing at those of the pure RPUF samples, which can be attributed to the abundant expanded graphite formed in the char during the cone test. Comparing the morphology of char residue at different apparent densities, due to the increase of apparent density, the char layer became more compact and retard the heat release effectively, and form the HRR behavior characteristics of thermally thick charring sample in Figure 7b. The char residues of RPUF/APP system at different apparent densities are shown in Figure 11. 

The morphologies of char residues of RPUF/APP were more compact compared at those of RPUF and RPUF/EG. However, no crack appeared on the surface of RPUF/APP at 30 kg/m^3^, while several cracks appeared on the surfaces of RPUF/APP at 60 and 90 kg/m^3^. Combining at the analysis in HRR curves from Figure 6c, the sample at 30 kg/m^3^ showed a sharp peak, while the samples at 60 and 90 kg/m^3^ showed two peaks (a sharp peak and a short peak). Therefore, the char layer formed during the combustion process of RPUF/APP at 60 and 90 kg/m^3^ broke by the release of congregated degradation products, which caused the appearance of the second short HRR peak in Figure 7c. 

### 3.5. Analysis of Flame Retardancy Mechanism

For all RPUF systems, the cell sizes decreased gradually at the increased apparent density from 30 to 90 kg/m^3^. RPUF as a kind of charring polymers, the smaller cell sizes were more beneficial to the cohesion of the cell wall after contraction during the combustion process. Therefore, the LOI value of RPUF increased slightly (the maximal increment of LOI was 0.5%) at the increase of apparent density. For RPUF/APP systems, the APP content in each of these three samples was 10 wt. %, therefore, at the same volume, the mass of APP in the sample at higher-density was more than that of the lower-density sample. As is well-known, APP is a kind of condensed phase flame retardant due to its dehydration and carbon-forming effect at the polymer matrix. It can be speculated that the sample at smaller cell sizes in the presence of APP was more beneficial to the cohesion of the cell wall to form the char layer. This can be evidenced by the increased maximal LOI increment (the maximal increment: 1% for RPUF/APP system (from 22.5% to 23.5%), 0.5% for RPUF system (from 20.5% to 21%)) from the LOI results. For RPUF/APP systems, more EG particles exist in the surface of the sample at higher apparent density can form a mass of expanded graphite sheet during the combustion process. A compact char layer can be formed on the high-density sample, which can enhance flame-retardant properties of RPUF/EG sample. This also can be evidenced by the remarkable increased maximal LOI increment (the maximal increment: 3% for RPUF/EG system (from 24% to 27%), 0.5% for RPUF system (from 20.5% to 21%)) from the LOI results.

## 4. Conclusions

The effect of apparent density on flame retardancy, thermal degradation, and combustion behaviors of RPUF, RPUF/EG and RPUF/APP were investigated in this paper. LOI results showed that the increase of apparent density is beneficial to the flame retardancy enhancement of RPUF, RPUF in the presence of APP or EG, especially EG. The results of TGA revealed that the influences of apparent density on the two degradation stages of RPUF, RPUF/EG and RPUF/APP systems are nearly contrary. The increase of apparent density of sample caused more weight loss in the first degradation stage and less weight loss in the second degradation stage in all these three systems. The TGA results also showed that the enhancement of APP or EG on thermal stability of RPUF is more significant under the conditions of higher apparent density. The cone calorimeter results indicated that the apparent density played an important role in the combustion performance of RPUF and RPUF filled by EG or APP. The duration of heat release and THR of RPUF, RPUF/EG and RPUF/APP increased gradually at the increase of apparent density. The combustion behavior characteristics changed from thermally thin materials to the thermally thick charring samples at the increase of apparent density from 30 to 90 kg/m^3^. Therefore, apparent density should be taken into account when investigating the effect of flame retardants on flame retardancy, thermal degradation, and combustion behaviors of RPUF.

## Figures and Tables

**Table 1:** Formulations of RPUF, RPUF/EG and RPUF/APP.

Sample	4110	A33	LC	Si-Oil	TEA	Water	PM-200	APP	EG
RPUF30	100	1	0.5	2	3	4.5	191.51	0	0
RPUF60	100	1	0.5	2	3	1.5	143.39	0	0
RPUF90	100	1	0.5	2	3	0.5	127.35	0	0
RPUF30/EG	100	1	0.5	2	3	6	215.58	36.45	0
RPUF60/EG	100	1	0.5	2	3	1.8	148.2	28.5	0
RPUF90/EG	100	1	0.5	2	3	0.8	132.16	26.6	0
RPUF30/APP	100	1	0.5	2	3	5.5	207.55	0	35.51
RPUF60/APP	100	1	0.5	2	3	1.8	148.2	0	28.5
RPUF90/APP	100	1	0.5	2	3	0.8	132.16	0	26.6

**Table 2:** TG/DTG data of RPUF, RPUF/EG and RPUF/APP under air atmosphere.

Samples	*T*_initial_^a^, ℃	Stage I		Stage II		Char Residue at700 ℃, %
		*T*_max_^b^, ℃	W_I_^c^, %	*T*_max_^b^, ℃	W_II_^c^, %	
RPUF30	274.14	330	41.99	562	54.83	3.18
RPUF90	271.61	332	50.67	561	49.19	0.14
RPUF30/EG	258.56	318	41.89	553	50.72	6.76
RPUF90/EG	263.69	316	44.71	558	42.87	10.17
RPUF30/APP	272.5	311	36.87	549	46.11	12.14
RPUF90/APP	274.26	304	40.97	539	38.36	14.94

a. *T*_initial_ is the initial degradation temperature (temperature at 5.0% weight loss). b. *T*_max_ is the maximum-rate degradation temperature. c. W_I_ or W_II_ is the weight loss at stage I or stage II.

**Table 3:** Cone data of RPUF, RPUF/EG and RPUF/APP at 35 kW/m^2^ heat flux.

Samples	pHRR (kW/m^2^)	THR (mJ/m^2^)
RPUF30	233.68	17.01
RPUF60	188.75	25.93
RPUF90	207.48	37.98
RPUF30/EG RPUF60/EG RPUF90/EG RPUF30/APP RPUF60/APP RPUF90/APP	100.06 114.71 112.52 195.35 144.34 151.86	12.94 23.63 30.78 10.76 20.41 34.87
